# Calcium supplementation for prevention of pre-eclampsia in high-risk women: study protocol for a randomised triple-blind placebo-controlled trial (CaPE)

**DOI:** 10.1186/s13063-026-09513-w

**Published:** 2026-03-18

**Authors:** Shireen Meher, Alisha Maher, Peter Brocklehurst, Lucy Chappell, Andrew Ewer, Marcus Green, Fadil M. Hannan, Kim Hinshaw, Max Hughes, Louise Jackson, Patrick Moore, Katie Morris, Rachel Plachcinski, Julia Sanders, Lee Middleton

**Affiliations:** 1 University of Sydney, Nepean Clinical School, Kingswood, NSW Australia; 2 University of Birmingham, Institute of Metabolism and Systems Science, Birmingham, UK; 3 Birmingham Womens and Childrens NHS Foundation Trust, Birmingham, UK; 4 Birmingham Clinical Trials Unit, University of Birmingham, Birmingham, UK; 5 Department of Women and Children’s Health, School of Life Course Sciences, King’s College London, London, UK; 6 College of Medical Sciences, University of Birmingham, Birmingham, UK; 7 Action on Pre-eclampsia Charity, Evesham, UK; 8 Nuffield Department of Women’s & Reproductive Health, University of Oxford, Oxford, UK; 9 Sunderland Royal Hospital, Sunderland, Tyne & Wear UK; 10 Health Economics Unit, University of Birmingham, Birmingham, UK; 11 Health Economics and Health Policy at Bristol, Bristol Medical School, University of Bristol, Bristol, UK; 12 National Childbirth Trust, London, UK; 13 School of Healthcare Sciences, Cardiff University, Cardiff, UK

**Keywords:** Pre-eclampsia, Hypertension, Calcium, Pregnancy, High risk, Randomised controlled trial

## Abstract

**Background:**

Pre-eclampsia is a multisystem disorder affecting 2.8% of pregnancies in the UK. It usually presents after 20-week gestation with new-onset high blood pressure and proteinuria. Complications include eclampsia, stroke, and HELLP syndrome for the mother and preterm birth, fetal growth restriction, and stillbirth for the baby. Delivery is the only definitive cure, with antenatal care focusing on early detection and management of complications and optimising timing of delivery. Previous studies have shown calcium supplementation may reduce the risk of pre-eclampsia, but findings are driven by large effects seen in small trials that have not been replicated in larger trials. Subgroup analysis suggests benefits may only be seen in women with low dietary calcium intake, so findings may not be applicable to populations with adequate dietary calcium. Although the largest benefits appear to be in high-risk women, data are very limited, with no large trials conducted.

**Methods:**

CaPE is a two-arm parallel triple-blinded, placebo-controlled, multicentre, superiority randomised controlled trial testing the hypothesis that in pregnant women at increased risk, calcium supplementation is effective in reducing the occurrence of pre-eclampsia. The study will recruit 7756 women from approximately 60 obstetric units in hospitals across the UK. Women with a confirmed viable pregnancy, with gestation 22 + 0 weeks or less and deemed eligible for aspirin therapy based on either NICE guideline criteria (at least one high-risk factor or two or more moderate risk factors) or the Fetal Medicine Foundation (FMF) algorithm will be eligible to be randomised in a 1:1 ratio to receive either 2 g per day of calcium supplementation or placebo taken from 12 to 22 weeks, up to birth. The primary outcome is clinician diagnosis of pre-eclampsia, based on the ISSHP definition. Key secondary outcomes are severe pre-eclampsia index and preterm birth < 37 weeks; other secondary outcomes include the Pre-eclampsia Core Outcome Set (COS).

**Discussion:**

Calcium supplementation in high-risk women is an attractive intervention due to its potential efficacy, low cost, and safety profile, but a definitive trial is required to confirm benefits.

**Trial registration:**

ISRCTN 12033893. Registered on 25 May 2021.

## Administrative information

Note: The numbers in curly brackets in this protocol refer to SPIRIT checklist item numbers. The order of the items has been modified to group similar items (see http://www.equator-network.org/reporting-guidelines/spirit-2013-statement-defining-standard-protocol-items-for-clinical-trials/).

**Table:** 

Title {1}	Calcium supplementation for prevention of pre-eclampsia in high-risk women: study protocol for a randomised placebo-controlled trial (CaPE)
Trial registration {2a and 2b}	EudraCT number: 2020–004435−25 ISRCTN12033893
Protocol version {3}	V2.0, 08 August 2024
Funding {4}	National Institute for Health and Care Research Health Technology Assessment programme
Author details {5a}	Author list and affiliations 1. Shireen Meher*^1,2,3^ 2. Alisha Maher*^4^ 3. Peter Brocklehurst^4^ 4. Lucy Chappell^5^ 5. Andrew Ewer^6^ 6. Marcus Green^7^ 7. Fadil M. Hannan^8^ 8. Kim Hinshaw^9^ 9. Max Hughes^4^ 10. Louise Jackson^10^ 11. Patrick Moore^11^ 12. Katie Morris^4^ 13. Rachel Plachcinski^12^ 14. Julia Sanders^13^ 15. Lee Middleton^4^ *Joint first author 1. University of Sydney Nepean Clinical School, NSW, Australia 2. University of Birmingham, Institute of Metabolism and Systems Science, Birmingham, UK 3.Birmingham Women's and Children's NHS Foundation Trust, Birmingham, UK 4. Birmingham Clinical Trials Unit, University of Birmingham, Birmingham, UK 5. Department of Women and Children’s Health, School of Life Course Sciences, King’s College London, UK 6. College of Medical Sciences, University of Birmingham, Birmingham, UK 7. Action on Pre-eclampsia, Charity, Evesham, UK 8. Nuffield Department of Women’s & Reproductive Health, University of Oxford, Oxford, UK 9. Sunderland Royal Hospital, Tyne & Wear, UK 10. Health Economics Unit, University of Birmingham, Birmingham, UK 11. Health Economics and Health Policy at Bristol, Bristol Medical School, University of Bristol, UK 12. National Childbirth Trust, London, UK 13. School of Healthcare Sciences, Cardiff University, UK
Name and contact information for the trial sponsor {5b}	University of Birmingham Email: researchgovernance@contacts.bham.ac.uk
Role of sponsor {5c}	The study funder has not been involved in the study design, writing of the report, or the decision to submit for publication.

## Introduction

### Background and rationale {6a}

Pre-eclampsia is a multisystem disorder affecting 2.8% of pregnancies in the United Kingdom (UK) [1]. It usually presents after 20 weeks of gestation with new-onset high blood pressure, proteinuria, and/or involvement of other maternal organs [2]. Severe complications include eclampsia, stroke, haemolysis elevated liver enzymes low platelets (HELLP) syndrome, and disseminated intravascular coagulation (DIC). Risks to the fetus are also increased and may present as fetal growth restriction and stillbirth [3]. Pre-eclampsia is a leading cause of iatrogenic prematurity [4]. It also leads to significant National Health Service (NHS) resource utilisation due to increased surveillance, hospital admissions, and higher-level care [5].

The exact cause of pre-eclampsia remains unclear but is thought to occur secondary to poor placentation and endothelial cell damage, leading to vasoconstriction, abnormal coagulation, and organ dysfunction [6]. Once diagnosed, delivery is the only definitive cure, with antenatal care focusing on early detection and management of complications, as well as optimizing timing of delivery. Preventative strategies, including antiplatelet agents (low-dose aspirin) and calcium, may be effective in reducing the risk of pre-eclampsia [5].

A Cochrane review of 77 trials (40,249 women and their babies) demonstrated that aspirin reduces pre-eclampsia risk by 18% [7], with modest benefits in reducing the risk of death in the baby, preterm birth, small for gestational age babies, and having a pregnancy with a serious adverse outcome in women with risk factors for pre-eclampsia. Aspirin may have greater benefits in reducing pre-eclampsia leading to birth prior to 37-week gestation [8]. However, aspirin may slightly increase the risk of bleeding complications including postpartum haemorrhage [9]. National Institute for Health and Care Excellence (NICE) and other international guidelines recommend low-dose aspirin (75–150 mg) from 12-week gestation for women with any high-risk factor or multiple moderate-risk factors for pre-eclampsia, and aspirin is now standard of care in the UK and other parts of the world [10].

Epidemiological studies suggest an inverse relationship between calcium intake and pre-eclampsia risk [11–14]. Calcium may lower blood pressure by reducing parathyroid hormone release and intracellular calcium, which leads to reduced vascular smooth muscle contractility and vasoconstriction and lower resistance in uterine and umbilical arteries, increasing placental perfusion and oxygenation to the baby [15, 16]. A Cochrane review of 14 trials (15,730 women) found calcium supplementation (1.5–2 g daily) reduced pre-eclampsia risk by 55% (*RR* 0.45, 95% *CI* 0.31–0.65), preterm birth by 24% (*RR* 0.76, 95% *CI* 0.60–0.97), and serious maternal morbidity and mortality by 20% (*RR* 0.80, 95% *CI* 0.65–0.97) [17]. Concerns about potential adverse effects, including renal calculi, have not been substantiated in clinical trials [17]. However, the benefits of calcium supplementation for pre-eclampsia may be limited to populations with low dietary calcium intake. Subgroup analysis showed marked risk reduction in women with low baseline dietary calcium intake (*RR* 0.36, 95% *CI* 0.20–0.65; 8 trials, 10,678 women) but not in those with adequate intake (*RR* 0.62, 95% *CI* 0.32–1.20; 4 trials, 5022 women). Trials recruiting high-risk women suggest a 78% reduction in pre-eclampsia risk (*RR* 0.22, 95% *CI* 0.12–0.42; 5 trials, 587 women), though studies are small, use varied definitions of high risk, and recruited women with both adequate and inadequate dietary calcium intake [17]. Large trials in high-risk populations are lacking.

Calcium supplementation for pre-eclampsia prevention is not currently recommended in the UK [10], as prior trials predominantly recruited low-risk women with low dietary calcium intake, so findings have not been viewed as applicable to the UK population. There is limited data on dietary calcium intake in pregnant women in the UK, but three small studies (total 215 women) suggest a mean intake from 720 to 1345 mg/day [18]. However, as calcium supplementation appears to be most beneficial for women at high risk of pre-eclampsia, an adequately powered, large randomised trial is needed to evaluate its efficacy in high-risk women, regardless of baseline dietary calcium intake. The importance of this research question has been recognised by NICE [10]. Calcium is an attractive intervention due to its low cost, safety, and potential for reducing maternal and perinatal morbidity. This research could lead to improved maternal and perinatal outcomes and NHS cost savings.

Combining calcium with aspirin may also have synergy through addressing both vascular dysfunction and coagulation abnormalities, the two primary pathological mechanisms seen in pre-eclampsia. A prior small trial assessing combined therapy was underpowered to detect meaningful differences, highlighting the need for further research [19].

### Objectives {7}

The primary objective is to test the hypothesis that in pregnant women at increased risk of pre-eclampsia, calcium supplementation given in a dose of 2 g per day during pregnancy plus usual care (including aspirin) is more effective than usual care alone in reducing the relative risk for the occurrence of pre-eclampsia by at least 20%.

The secondary objectives are as follows:

1. To assess the impact of calcium supplements on other important outcomes for the woman and baby
2. To assess the cost-effectiveness of calcium plus usual care compared to usual care alone
3. To assess whether calcium has a differential effect in pre-specified subgroups of women
4. To assess the degree to which pregnant women are able to adhere to a calcium supplementation regimen

## Methods: participants, interventions, and outcomes

### Trial design {8}

The CaPE trial is a parallel-group, triple-blinded, placebo-controlled, multicentre, two-arm superiority randomised controlled trial with a 12-month internal pilot and health economics evaluation. Participants will be randomised at the level of the individual in a 1:1 ratio to either the equivalent of 2 g per day of calcium supplementation or placebo starting from between 12^+0^ and 22^+0^ weeks gestation and continuing up to delivery. The primary outcome will assess the superiority of calcium supplementation at reducing the risk of women developing pre-eclampsia.

#### Study setting {9}

The study will be conducted in up to 60 obstetric units in hospitals across the UK. A list of participating study sites can be found on the CaPE trial website: https://www.birmingham.ac.uk/research/bctu/trials/womens/cape/recruitment.

#### Eligibility criteria {10}

Various screening strategies to identify women at high risk of pre-eclampsia have been explored [20]. To date, NICE clinical risk factor screening, as listed below, remains the standard of care nationally for identification of high-risk women eligible for aspirin therapy in early pregnancy. A limited number of hospitals in the UK use the Fetal Medicine Foundation (FMF) algorithm to screen for risk of pre-eclampsia for aspirin eligibility [21]. In addition to the maternal risk factors included in NICE guidelines, the algorithm uses biophysical (mean arterial pressure, uterine artery doppler) and biochemical (placental growth factor, PAPP-A) markers; this algorithm has been found to have a better detection rate for pre-eclampsia and preterm pre-eclampsia compared to the NICE criteria for a 10% screen-positive rate [22]. However, as pre-eclampsia screening is an evolving area of research and national recommendations may change during the course of the trial, as a pragmatic approach, we will include women deemed to be eligible for aspirin based on NICE, FMF, or any other nationally accepted risk assessment tool. This will ensure findings will remain generalisable and clinically relevant.

There may be some women who are deemed eligible for aspirin but cannot take it due to contraindications. These women are still eligible to take part in CaPE and are particularly important to assess for calcium benefits.

##### Inclusion criteria

- 16 years of age or older
- Able to provide informed consent.
- Confirmed viable pregnancy on a dating scan (usually done between 10 and 14 weeks) and any subsequent scans
- Gestation 22^+0^ weeks or less
- Women deemed eligible for aspirin therapy based on the following:
  1. NICE guideline criteria as follows:
    Either at least one high-risk factor
    Hypertensive disease during a previous pregnancy
    Chronic renal disease
    Autoimmune disease such as systemic lupus erythematosus (SLE) or antiphospholipid syndrome
    Type 1 or 2 diabetes
    Chronic hypertension
    or two or more moderate-risk factors
    Nulliparity
    Age 40 years or older
    BMI 35 kg/m^2^ or more at first visit
    Family history of pre-eclampsia
    Multifetal pregnancy
    Pregnancy interval of more than 10 years
    OR
  2. The Fetal Medicine Foundation (FMF) algorithm for pre-eclampsia risk assessment [21] OR
  3. Any other national pre-eclampsia screening criteria guidelines that may be used in the future

##### Exclusion criteria

- Any known contraindications to regular calcium intake (history of renal stones, known renal impairment with pre-pregnancy eGFR < 30 mL/min/1.73 m^2^ or serum creatinine > 150 μmol/L, known history of hypercalcaemia or hypercalcaemia-causing diseases (e.g. parathyroid disease, sarcoidosis, malignancy)) and current severe persistent vomiting leading to dehydration or requiring hospitalisation*.
  *If persistent vomiting resolves, the woman may be reassessed for inclusion in the trial, providing all other inclusion and exclusion criteria are met.
- Use of drugs with potential for severe interactions with calcium: Digoxin or other cardiac glycosides, antiretroviral drugs for HIV treatment, antineoplastic drugs, and diuretics (thiazide, thiazide-like, or xipamide)—the latter two are not usually used in pregnancy [23]
- Use of any additional calcium supplement either on its own or as part of other multivitamin or vitamin D preparations and unable or unwilling to stop them or change to other multivitamins without calcium, as this could lead to higher doses of calcium supplementation in the calcium group and contamination in the placebo group
- Women who are taking vitamin D regularly in high doses > 1000 IU/day as supplements or for conditions such as malabsorption syndromes. Note: A short course of high-dose vitamin D (e.g. 20,000 IU weekly for 6 weeks) to treat vitamin D deficiency during pregnancy is not an exclusion criterion
- Known contraindications to excipient isomalt (e.g. hereditary fructose intolerance)
- A diagnosis of pre-eclampsia in the current pregnancy, prior to trial entry

#### Who will take informed consent? {26a}

Women who are eligible will be approached by a member of the direct care team, which may include the maternity research team. Information about the trial will be provided both verbally and in writing via the participant information sheet (PIS). Women will be given time to consider participation in the trial and ask any questions they may have. If the women would like to take part in the trial, then a doctor trained in study procedures will confirm eligibility, and an appropriately trained (including good clinical practice (GCP) trained) individual will be able to take consent, providing that local practice allows this and responsibility has been delegated by the site principal investigator (PI). The participant’s study participation will be documented in the paper or electronic maternity records.

## Additional consent provisions for collection and use of participant data and biological specimens {26b}

The consent form states that participants will allow direct access to the medical records of the mother and baby if they agree to take part in the trial. Optional consent is available to take part in future research related to the study via electronic data linkage of routinely collected data from NHS databases and general practitioner records.

Consent for biological specimens is not applicable as no samples are being collected.

### Interventions

#### Explanation for the choice of comparators {6b}

The current standard of practice is not to give calcium supplementation; hence, no treatment aside from usual care is our comparator. We will use placebo tablets that contain no active ingredient and are manufactured to match the chewable tablets that are given to the intervention arm. The rationale for using a placebo is described in the section ‘Who will be blinded {17a}’. Participants are instructed to take one tablet in the morning and one in the evening.

### Intervention description {11a}

Participants randomised to the intervention arm will receive 2 g/day of calcium supplementation through oral, chewable tablets containing the equivalent of 1 g of calcium carbonate in each tablet. Participants will be instructed to take one tablet in the morning and one in the evening.

However, if the participant in the intervention or comparator arm is unable to take tablets in divided doses, they will be given the option to take both tablets at a more convenient frequency (i.e. at the same time) to improve adherence but will be advised of potential increase in risk of gastrointestinal side effects.

The dose of 2 g/day has been selected for this study to ensure the highest likelihood of detecting a difference in the risk of pre-eclampsia if one exists by using a high dose of calcium, without there being any known risks for women and their babies, based on the best available evidence from previous studies. Previous trials suggest a dose-dependent effect: a dose of 2 g/day is the highest, and most used dose among previous RCTs that show a significant reduction in the risk of pre-eclampsia [17]. One RCT directly comparing high-dose calcium (2 g/day) with low dose (500 mg/day) found a greater reduction in the risk of pre-eclampsia with 2 g/day [24], although a non-inferiority trial comparing 1.5 g/day with 500 mg/day did not find a significant difference [25]. The dose of 2 g/day administered in this trial is within the maximum daily intake unlikely to cause adverse health effects (tolerable upper intake level), which is around 2.5 to 3 g/day (for 19–50 and 14–18 years, respectively) during pregnancy [26]. A previous large randomised trial using a dose of 2 g/day in pregnant women with adequate baseline calcium intake (> 1 g/day) showed that it was not associated with any increase in adverse effects [27]. Thus, we aim to optimise efficacy without compromising safety, based on the best available evidence, and ensure the greatest likelihood of a definitive result for the trial.

The optimum time of starting calcium is unclear as the mechanism of action is not clearly understood. Over 90% of women in previous calcium supplementation trials were randomised at or prior to 22 weeks [17]. In line with previous trials, CaPE IMP may be started anytime from between 12 and 22 weeks. This will avoid IMP exposure in the first trimester, which is a time of greater teratogenic risk and randomisation at a time of greater pregnancy loss. Starting in the second trimester will also improve adherence as nausea and vomiting subside with advancing gestation. It is still early enough to potentially benefit women who are at risk of developing early onset pre-eclampsia. This timing will also coincide with routine antenatal appointments (dating and anomaly scan), making it easier and more efficient for clinicians to recruit and less time-consuming and inconvenient for women.

### Criteria for discontinuing or modifying allocated interventions {11b}

Discontinuation of the investigational medicinal product (IMP) may occur at either the request of the participant or clinician. The time and reason for discontinuation of the IMP will be documented, but unless the participant also expresses a desire to withdraw, they will remain in the trial and their outcome data collected. Similarly, if a clinical decision is made to discontinue a participant’s IMP usage, this will be documented, but in and of itself is not a reason to withdraw the participant.

Participants can withdraw from the trial at any time without giving a reason, and this will not impact their own (or their baby’s) ongoing care and standard clinic visits.

### Strategies to improve adherence to interventions {11c}

Adherence to the IMP will be captured through two methods: (1) A pill count where unused IMP is returned to the hospital at the end of pregnancy and (2) text messages to the participant enquiring about adherence to the IMP sent on a four-weekly basis. A ‘maintaining contact’ text message is also sent out monthly (2 weeks after the adherence text messages) to thank the participant for their time in the trial and to remind them to take their tablets as advised by their local care team. As a pragmatic trial, the regularity of these text messages is aimed at mimicking the frequency with which routine care contact may occur with the participant, both via clinic appointments and general healthcare phone calls and text messages which now form part of routine practice.

### Relevant concomitant care permitted or prohibited during the trial {11d}

Women commonly use other supplements or medications during their pregnancy that may impact or be impacted by calcium [23]. The common ones include the following:

- Iron: Calcium can decrease iron absorption. Women will be advised to take the trial tablets more than 2 h before or after any iron tablets.
- Vitamin D: Vitamin D impacts calcium absorption, and regular high doses may result in too much calcium in the blood (hypercalcaemia). For the CaPE trial, women can take part if they are on vitamin D supplements in a preventative dose (≤ 1000 IU) or short-term treatment dose of vitamin D (e.g. 20,000 IU weekly for 6 weeks) as it would be inappropriate to withhold these. Women will be asked not to use vitamin D supplements containing additional calcium. The use of or intention to use vitamin D will be recorded at the start of the pregnancy to confirm eligibility.
- Multivitamins: During pregnancy, women may routinely take multivitamins; however, whilst in the CaPE trial, they should not take multivitamins containing calcium, as this may lead to hypercalcaemia in the intervention arm or contamination of the placebo arm.
- Antacids: women will be advised to take non-calcium-based antacids, such as aluminium or magnesium-based antacid, to reduce the risk of hypercalcaemia. Proton-pump inhibitors (such as omeprazole) can also be used as alternatives.
- Thyroxine and hydroxychloroquine: Calcium may reduce their absorption, so women will be advised to leave a period of at least 4 h before taking their trial tablet if they use these medications.
- Folic acid: No known interaction with calcium, so women may take this as normal.

Women will be given a drug information sheet at the time of recruitment, which details what supplements and medications are suitable to take at what time point relative to the IMP. Otherwise, participants can receive concomitant care as clinically required.

Co-enrolment in other randomised controlled trials may be acceptable but must be discussed and agreed upon by the chief investigator (CI) and Trial Management Group (TMG).

### Provisions for posttrial care {30}

Trial outcomes will be collected until primary hospital discharge after birth or 28 days after estimated delivery date (EDD), whichever is sooner. IMP will be stopped at the end of pregnancy, so any postnatal data is unlikely to reflect the effect of the intervention. Postnatal care will be determined by the woman’s usual care team. Serious adverse event (SAE) data on maternal and neonatal death will be collected up to 28 days after discharge if the research team become aware of any such events.

### Outcomes {12}

The following outcomes are collected from randomisation to maternal and neonatal hospital discharge or up to 28 days after EDD, whichever is sooner.

#### Primary outcome

The primary outcome of the CaPE trial is a clinician diagnosis of pre-eclampsia, based on the International Society for the Study of Hypertension in Pregnancy (ISSHP) definition: a blood pressure ≥ 140/90 mmHg (at least two readings several hours apart) and either.

1. Significant proteinuria (protein/creatinine ratio (PCR) of 30 mg/mmol or more) OR
2. Maternal multiorgan dysfunction as follows:
  - Acute kidney injury (AKI) (creatinine ≥ 90 μmol/L)
  - Liver involvement (elevated transaminases, e.g. alanine transaminase (ALT), or aspartate transaminase (AST) > 40 IU/L) with or without right upper quadrant or epigastric abdominal pain).
  - Neurological complications (including eclampsia, altered mental status, blindness, stroke, clonus, severe headaches, persistent visual scotomata)
  - Haematological complications (thrombocytopenia — platelet count below 150,000/μL, DIC, haemolysis).
  OR
3. Uteroplacental insufficiency (foetal growth restriction, abnormal umbilical artery Doppler, stillbirth) developing at or after 20-week gestation

#### Key secondary outcomes

The following are considered key secondary outcomes for the analysis as they are clinically very important and have a potential to be impacted by calcium based on data from previous trials as follows:

- Severe pre-eclampsia index: any one of severe pre-eclampsia, early onset pre-eclampsia < 32 weeks, eclampsia, placental abruption, HELLP, or severe gestational hypertension [28]
- Preterm birth < 37 weeks

#### Other secondary outcomes

##### For the woman

Pre-eclampsia Core Outcome Set (COS) outcomes, namely:

- Death
- Eclampsia
- Stroke
- Visual impairment: retinal detachment or cortical blindness
- Pulmonary oedema
- Acute kidney injury: creatinine ≥ 90 μmol/L
- Liver capsule haematoma or rupture (confirmed on ultrasound)
- Raised liver enzymes: ALT or AST > 40 IU/L
- Low platelets < 150,000/μL
- Abruption
- Postpartum haemorrhage: estimated or measured blood loss ≥ 500 mL and ≥ 1000 mL after birth
- Admission to intensive therapy unit (ITU)
  o Any admission
  o Days of admission
- Use of mechanical ventilation (for other than caesarean section)

In addition to these COS outcomes as follows:

- Gestational hypertension: new onset hypertension ≥ 140/90 mm Hg (at least two measurements several hours apart) after 20-week gestation in the absence of proteinuria or other features of pre-eclampsia
- Severe hypertension: blood pressure ≥ 160 systolic and/or 110 mm Hg diastolic
- Severe gestational hypertension: new onset hypertension ≥ 160 mm Hg systolic and/or 110 mm Hg diastolic after 20-week gestation in the absence of proteinuria or other features of pre-eclampsia
- Severe pre-eclampsia, defined as pre-eclampsia with severe features (American College of Obstetricians and Gynecologists definition) including severe hypertension or low platelets < 100,000/μL, abnormal liver function tests (liver enzymes at least twice the upper limit of normal) and right upper quadrant pain not accounted for by other diagnoses, abnormal renal function (creatinine > 1.1 mg/dL), pulmonary oedema, visual impairment, severe headache unresponsive to medication, and no other cause found.
- HELLP syndrome based on a clinician’s diagnosis, supported by low platelets and raised liver enzymes as defined above with or without evidence of haemolysis (raised lactate dehydrogenase enzyme or blood film)
- Early onset pre-eclampsia < 32 weeks
- Pre-eclampsia requiring delivery before 37 weeks
- Use of magnesium sulphate for pre-eclampsia
- Onset of birth: spontaneous, induction of labour, or caesarean section
- Mode of birth: spontaneous vaginal birth, assisted vaginal birth, elective pre-labour caesarean section, emergency pre-labour caesarean section, and emergency caesarean section in labour
- Adverse effect: new diagnosis of maternal hypercalcaemia
- Adverse effect: renal stones (confirmed on imaging, after starting IMP)
- Adverse effect: stopping of medication due to adverse effects.

##### For the baby

COS outcomes, namely:

- Any death in the baby up to hospital discharge. Data will be collected separately for the following:
  o Fetal loss < 24-week gestation (miscarriage)
  o Fetal loss ≥ 24-week gestation (stillbirth)
  o Neonatal death (from birth up to 28 days)
    Early neonatal death (up to 7 days after birth)
    Late neonatal death (from 7 days to 28 days)
  o Perinatal death: stillbirth or neonatal death up to 7 days
  o Termination of pregnancy
- Gestational age at delivery (median, < 28 weeks, < 32 weeks, < 37 weeks)
- Birthweight (mean, < 3rd centile, < 10th centile)
- Admission to neonatal unit (NNU)
  o Any admission
  o Level of neonatal care
  o Days of admission
- Respiratory support morbidity
  o Use of surfactant
  o Use of mechanical ventilation
  o Use of noninvasive ventilation
  o Use of supplementary oxygen
  o Duration of respiratory support
- Neonatal seizures
- Neonatal brain injury as follows:
  o Hypoxic ischaemic encephalopathy requiring therapeutic hypothermia
  o Neonatal stroke
  o Severe intraventricular haemorrhage (IVH) grade III/IV and/or cystic periventricular leukomalacia

In addition to these COS outcomes as follows:

- Chronic lung disease (CLD) requiring oxygen therapy at 36-week post-menstrual age
- Necrotising enterocolitis (NEC) requiring surgery
- Retinopathy of prematurity (ROP) requiring treatment with laser or anti-VEGF injection
- Composite of death or serious morbidity: Death or CLD, IVH grade III/IV, NEC requiring surgery, or ROP requiring treatment
- Adverse effects: Neonatal hypocalcaemia requiring treatment

Whilst the outcomes listed above will allow us to capture all events relevant to the trial’s main question on prevention of pre-eclampsia, we will seek consent from trial participants to be approached for long-term data linkage studies in the future (requiring additional and separate funding and ethical approval). This could include assessment of cardiovascular health for both women and babies.

#### Participant timeline {13}

**Fig. 1:**
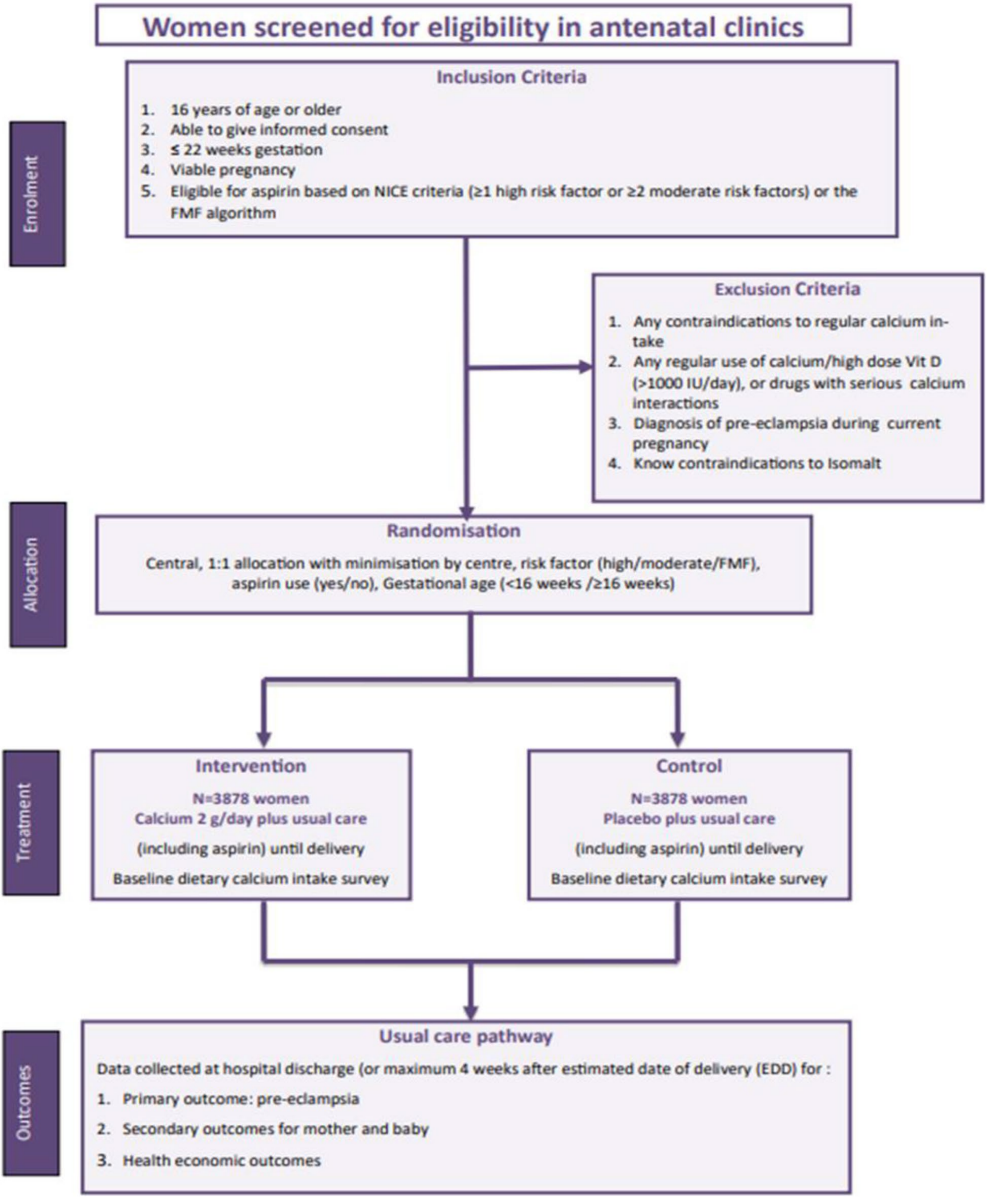
Participant flow through CaPE trial

Participant timeline is presented in Fig. 1 and Table 1.

**Table 1:** Schedule of assessments

				From randomisation		
Visit	Screening	Baseline	After recruitment — as identified	Every 4 weeks until delivery	Admission for delivery	Hospital discharge/estimated delivery date plus 4 weeks
Eligibility check	x	x				
Dietary calcium intake survey		x				
Intention to take or taking vitamin D		x				
Intention to take or taking aspirin		x				
Valid informed consent		x				
Randomisation		x				
Dispensing of IMP		x				
Adherence text message (automated)				x		
Outcome CRF Completion						x
Serious adverse events (SAE)			x			x *
Return unused IMP (pill counting)					x**	

*Any events meeting the trial definition of a reportable SAE should be reported to the CaPE trial office within 24 h of the site becoming aware. SAE reporting begins from the commencement of trial treatment and ends at hospital discharge after birth, 28 days after EDD, death, and complete withdrawal or lost to follow-up, whichever occurs sooner. The only caveat to this is in regard to death of the participant or neonate — this must be reported up to hospital discharge following birth or up to 28 days after EDD, whichever occurs later. Should a participant completely withdraw or be lost to follow-up, the requirement to report death will halt on that date

**At point of admission for delivery or as close to as possible

#### Sample size {14}

The trial will recruit women at high risk of pre-eclampsia, deemed eligible for aspirin based on NICE guideline criteria which includes a combination of (1) women with a single high-risk factor and (2) women with two or more moderate risk factors. The control group event rate of pre-eclampsia in women with a single high-risk factor on aspirin has been estimated to be 15% from the PARIS individual participant data (IPD) meta-analysis of antiplatelet agents for prevention of pre-eclampsia [29]. For women with two or more moderate risk factors, the control rate is estimated to be 8% from a retrospective study conducted at a tertiary referral unit [30]. We estimated the ratio of the two risk factor groups to be approximately 50:50 [31], giving an overall control group event rate of 11.5%.

*N* = 7756 women will be recruited (approximately 3878 in each group). This allows us to detect with 90% power (*p* = 0.05) a 20% relative risk reduction in pre-eclampsia from 11.5% down to 9.2%, allowing for a 5% loss to follow up (total 7368 women after attrition). A 20% reduction is considered plausible given the 55% reduction observed in the Cochrane review [17], albeit in a population more heterogeneous and distinct from that considered in CaPE. It is also likely to be considered clinically meaningful by clinicians and policymakers, given aspirin was adopted into clinical practice based on a 17% reduction [7]. The sample size was calculated using the power twoproportions command in Stata 18.

#### Recruitment {15}

Recruitment will happen from maternity hospitals across the UK. All pregnant women are routinely screened for aspirin eligibility for prevention of pre-eclampsia, generally at their booking appointment, either in the hospital or in the community. Trial information will be available in the hospital and/or community antenatal clinics (including leaflets, PISs, posters). In addition, CaPE information will be publicly available via online content, including social media, participating hospital websites, the CaPE website (including contact details of the local research team when provided), patient and public involvement (PPI) websites/forums, etc.

## Assignment of interventions: allocation

### Sequence generation {16a}

Randomisation will be conducted through a secure randomisation system hosted at the University of Birmingham. Participants will be randomised in a 1:1 ratio to either calcium or placebo. A minimisation algorithm will be used within the online randomisation system to ensure balance in the treatment allocation over the following variables:

- Trial site
- Risk factor for pre-eclampsia (one or more high risk factors/two or more moderate risk factors/high risk on the FMF algorithm)
- Intention to use aspirin for prevention of pre-eclampsia (yes/no)
- Gestational age at randomisation (< 16 weeks/≥ 16 + 0 weeks)

Women may have a high-risk factor along with moderate risk factors; in this case, women will be minimised based on the high-risk factor as they are likely to be in the group at highest risk of developing pre-eclampsia. Where the FMF algorithm has been used to ascertain high risk for pre-eclampsia, women will be minimised based on the use of the FMF algorithm regardless of whether they have high or moderate risk factors based on NICE criteria, as women who are screen positive on the FMF algorithm have a higher probability of developing pre-eclampsia compared to the use of the NICE criteria risk factors alone. A random element will be included in the minimisation algorithm so that each participant has a probability of being randomised to the opposite treatment that they would have otherwise received.

### Concealment mechanism {16b}

Randomisation will be performed by a central secure online randomisation system hosted by Birmingham Clinical Trials Unit (BCTU) (available at http://www.trials.bham.ac.uk/cape), ensuring allocation sequence is concealed.

### Implementation {16c}

The allocation sequence is generated by the secure online randomisation system. Women who are enrolled in the trial will be entered into the randomisation system by trained healthcare professionals who have been delegated the role of randomising participants by the site’s PI. The randomisation system assigns the participant to either calcium or placebo using the minimisation algorithm described in the section ‘Sequence generation {16a}’.

## Assignment of interventions: blinding

### Who will be blinded {17a}

This will be a triple-blind placebo-controlled trial. Allocation concealment is preserved at the manufacturing level, so the trial participants, their medical/care team, and research team/investigators (including the statistician) will be unaware of the treatment allocation (either calcium or placebo). Blinding will prevent any participant and researcher bias and ensure valid results.

### Procedure for unblinding if needed {17b}

Authorised members of the research team can be unblinded to treatment allocation in case of a medical emergency and/or SAE where the information about the woman’s treatment allocation is required for the continued medical management of the woman or her child. Unblinding is anticipated to be a rare event. This can be done via the CaPE database (if delegated the duty by their PI) or by contacting the CaPE trial office. Any unblinding will be monitored centrally.

For reporting purposes to regulatory bodies, cases that are considered serious, unexpected, and possibly, probably, or definitely related will be unblinded at the trial office by the trial manager (or other nominated individual). Members of the local care team and the participant will not be made aware of the actual trial treatment allocation unless it is deemed clinically necessary for the ongoing care of the participant by the CI or the local clinical team. In all other circumstances, the participant, the investigators, and research midwives/nurses will remain blind to treatment allocation, whilst the participant remains in the trial.

### Data collection and management

#### Plans for assessment and collection of outcomes {18a}

A summary of the data being collected is provided in Table 1. Prior to opening to recruitment, each site participating in CaPE will receive training on the protocol and data collection procedures during the site initiation visit. Once an eligible participant has provided informed consent, baseline data (including intention to take vitamin D, intention to take aspirin, and risk factors for pre-eclampsia) will be recorded within the randomisation data collection form by the site research team. Participants will be required to complete a short, validated dietary calcium intake questionnaire [32] immediately after randomisation, and their daily baseline dietary calcium intake will be calculated via a web-based calculator [33] and recorded. Participants will remain blinded to these results to avoid influencing their dietary behaviour. All outcome data will be captured on the electronic case report forms (CRFs) available through the CaPE database. This data will be collected from standard maternity notes (both electronic medical records and paper-based records), by case note review, at primary hospital discharge or 28 days after EDD, whichever is sooner. Plans for data quality and monitoring are reported in the section ‘Frequency and plans for auditing trial conduct {23}’.

#### Plans to promote participant retention and complete follow-up {18b}

Participants will be provided with contact details for the local research team if they have any questions or wish to provide any additional information about their participation in the trial. A ‘maintaining contact’ text message will be sent automatically on a monthly basis (2 weeks after the adherence text message) to remind women to take their tablets as advised by their local care team, as well as thanking them for taking part in the trial. An automated text message reminder will also be sent close to EDD (~36-week gestation) asking women to return unused IMP back to the hospital at the end of their pregnancy. The research team may also communicate with the participants via text or phone calls to remind women to bring the IMP bottles back at the end of the pregnancy or respond to any questions from the participants.

### Data management {19}

CaPE trial data will be entered by sites to a bespoke BCTU trial database and is stored according to GCP guidelines. Processes pertaining to data accuracy will be detailed in the trial-specific data management plan. Coding and validation must be agreed between the trial manager, statistician, and programmer, and the trial database will be signed off only once the implementation of these has been agreed.

### Confidentiality {27}

Personal data recorded on all documents will be regarded as strictly confidential and is handled and stored in accordance with the General Data Protection Regulation, 2018 (and any subsequent amendments). Participants will always be identified using their unique trial identification number on the CRFs and on any correspondence between members of the BCTU and site research team.

BCTU will maintain the confidentiality of all participants’ data and will not disclose information by which participants may be identified to any third party other than those organisations for which the participant has given explicit consent for data transfer (e.g. the transfer of their mobile phone number to text local so the participant can receive texts measuring their adherence) or those who need access to fulfil regulatory or legal functions. Representatives of the CaPE trial team and sponsor may be required to have access to participants’ notes for quality assurance purposes, but participants will be reassured that their confidentiality will be respected at all times.

### Plans for collection, laboratory evaluation, and storage of biological specimens for genetic or molecular analysis in this trial/future use {33}

No biological samples will be collected.

## Statistical methods

### Statistical methods for primary and secondary outcomes {20a}

The separate statistical analysis plan (SAP) provides a more comprehensive description of the planned statistical analyses. A brief outline of these analyses is given below.

The primary comparison groups will be composed of those randomised to usual care plus an additional dietary calcium supplement of 2 g per day versus those randomised to usual care plus a placebo (the randomised groups).

In the first instance, all analyses will be based on the intention to treat principle, i.e. all participants will be analysed in the treatment group to which they were randomised, irrespective of adherence or other protocol deviation. For all outcome measures, appropriate summary statistics will be presented by group (e.g. proportions/percentages, mean/standard deviation, or median/interquartile range). All outcomes will be presented with point estimates (e.g. relative risks, incident rate ratios, hazard ratios, mean differences) and 95% confidence intervals. The final analysis dataset will only include items up to and including hospital discharge of the mother and baby or 28 days after EDD (whichever occurs sooner), with the exception of SAE data on maternal and neonatal death which will be collected up to 28 days after discharge if the research team become aware of any such events. Analyses will be conducted using complete case analysis, with participants excluded from the intention-to-treat analysis if their outcome data is missing.

#### Primary outcome

A mixed-effects log-binomial regression model will be used to calculate the risk difference and relative risk with 95% confidence intervals for the primary outcome (defined in the ‘Outcomes {12}’ section) adjusting for the minimisation variables listed in the ‘Sequence generation {16a}’ section. The *p*-value relating to the intervention group parameter as generated by the model estimating the relative risk will also be presented.

#### Secondary outcomes

Secondary outcomes are defined in the ‘Outcomes {12}’ section. Secondary maternal outcomes are all dichotomous (e.g. eclampsia occurred or not) and will be analysed using risk ratios and 95% confidence intervals, generated using a log-binomial regression model, adjusting for the minimisation variables listed in the ‘Sequence generation {16a}’ section. Dichotomous secondary outcomes for the baby (e.g. small for gestational age < 10th centile) will be analysed in the same fashion. Continuous outcomes for the baby (e.g. birthweight) will be analysed using a linear regression model, adjusting for the same factors to obtain a mean difference between groups, and a 95% confidence interval. Secondary outcomes will not be subject to hypothesis testing, and confidence intervals will be interpreted cautiously given the potential for multiplicity.

A trial-based health economic evaluation will be undertaken to explore the cost-effectiveness of the intervention to prevent pre-eclampsia in expectant mothers compared to standard care. The cost differences between the intervention and control groups will be measured, valued, and combined with the clinical effectiveness data, and the main analysis will assess the cost per case of pre-eclampsia prevented. In line with existing recommendations, the economic analysis will adopt a health care perspective by considering costs incurred by the NHS [34]. The analysis will use the individual-level data on resource use collected during the trial, and unit costs will be applied. The results will be presented initially in a disaggregated format using a cost consequences analysis and secondly using cost-effectiveness acceptability curves (CEAC) to reflect decision uncertainty across different thresholds of willingness to pay per additional unit of outcome. Deterministic and probabilistic sensitivity analyses will be undertaken to explore the robustness of the findings to plausible variations in key assumptions and analytical methods used and to consider the broader issue of generalisability of the trial’s results. The economic evaluation will be conducted and reported in line with relevant guidance [34–36].

### Interim analyses {21b}

Interim analyses of safety and efficacy for presentation to the independent Data Monitoring Committee (DMC) will take place during the trial. The committee has agreed on the manner and timing of such analyses and includes the analysis of the primary and major secondary outcomes, the primary outcome event rate, and full assessment of safety (SAEs) at least at annual intervals. Criteria for stopping or modifying the trial based on this information will be ratified by the DMC. Details of the agreed plan are written into the SAP and DMC charter.

### Methods for additional analyses (e.g. subgroup analyses) {20b}

Subgroup analyses are restricted to the primary outcome only. Adequate vs inadequate baseline dietary calcium intake (defined as ≥ 700 mg/day vs < 700 mg/day) is the main subgroup of interest, based on the hypothesis that calcium may be more efficacious in those women with inadequate intake.

Analysis will be carried out using a test for statistical heterogeneity, i.e. by including the treatment group by subgroup interaction parameter in the regression model to produce a *p*-value, and 95% confidence intervals will be produced for estimates within each subgroup and will be presented using forest plots.

Other subgroup analyses will include the following (full details to be listed in the SAP):

- At least one high risk factor for pre-eclampsia vs. two or more moderate risk factors
- Women deemed eligible based on NICE criteria vs FMF algorithm
- Gestational age at randomisation (< 16 weeks vs ≥ 16 + 0 weeks)
- Aspirin intake of 150 mg vs aspirin intake of 75 mg vs no aspirin
- Vitamin D supplement vs. no vitamin D supplement

Analyses will be considered exploratory and performed in the same manner as the main subgroup of interest, but confidence interval widths will be interpreted cautiously given the potential for multiplicity.

### Methods in analysis to handle protocol non-adherence and any statistical methods to handle missing data {20c}

Every attempt will be made to collect full follow-up data on all participating women and their babies; it is anticipated that missing data will be minimal. Participants with missing primary outcome data will not be included in the primary analysis in the first instance. This presents a risk of bias, and sensitivity analyses will be undertaken to assess the possible impact of the risk. This will include imputing missing data using multiple imputation techniques. Further sensitivity analysis will include an assessment of efficacy with those who had good adherence to treatment using a complier average causal effects (CACE) approach. Full details are included in the statistical analysis plan.

### Plans to give access to the full protocol, participant-level data, and statistical code {31c}

Access to the full trial protocol, statistical code, and an anonymised final trial dataset can be made available to external researchers upon request and with approval from the TMG and the BCTU data sharing committee in line with standard data sharing practices for clinical trial data sets.

## Oversight and monitoring

### Composition of the coordinating centre and trial steering committee {5d}

The trial coordinating centre is the Birmingham Clinical Trials Unit (BCTU), based at the University of Birmingham. The TMG will monitor all aspects of the conduct and progress of the trial as well as take responsibility for the day-to-day management of the trial. The TMG includes (but is not limited to) the CI, co-applicants, senior statistician, team leader, senior trial manager, health economist, and PPI representatives.

The TMG will report to the Trial Steering Committee (TSC), who will meet at least annually to provide trial oversight to ensure that the trial is running in a way which is both safe for the participants and provides appropriate feasibility data to the sponsor and investigators. TSC members include an independent chair, three other independent members (including a statistician), a PPI representative, an independent health economist, and the CI.

### Composition of the Data Monitoring Committee and its role and reporting structure {21a}

The DMC is independent from the sponsor and any competing interests and has been established to assess trial progress and important safety data. Data analyses will be provided in confidence to the DMC, and they will assess the safety and efficacy of the IMP during the trial and monitor the overall conduct of the clinical trial.

The DMC will operate in accordance with the trial-specific charter. The DMC will meet at least annually as agreed by the committee and documented in the charter. More frequent meetings may be required for a specific reason (e.g. safety) and will be recorded in the meeting minutes. The DMC may recommend stopping the trial early if there is ‘proof beyond reasonable doubt’ of clear benefit or harm of a treatment.

The DMC will report directly to the TSC, who will convey the recommendations of the DMC to the TMG. They will be asked to give advice on whether the accumulated data from the trial, together with the results from other relevant research, justifies the continuing recruitment of further participants.

### Adverse event reporting and harms {22}

CaPE sites will be required to report any adverse events (AEs) throughout their participation in the trial, from the day of commencement of trial treatment until primary hospital discharge after birth or 28 days after EDD, whichever is sooner. The only caveat to this is death of the participant or neonate — this must be reported up to hospital discharge following birth or up to 28 days after EDD, whichever occurs later. The recording and reporting of AEs will be in accordance with the UK Policy Framework for Health and Social Care Research, the Principles of GCP as set out in the UK Statutory Instrument (2004/1031 and subsequent amendments), and the requirements of the Health Research Authority (HRA) and The Medicines for Human Use (Clinical Trials) Regulations 2004 and amendments thereof.

AEs are rarely encountered in participants taking additional dietary calcium, and previous trials in pregnant women have consistently shown no increase in AEs. As the safety profile of the IMP used in this trial is well characterised and mild side effects have significant overlap with pregnancy symptoms (nausea, dyspepsia, abdominal pain, itching, etc.), we will only be reporting AEs that have a higher probability of being related to calcium intake on the outcome CRF. These include the following:

### Reportable adverse events

- New diagnosis of maternal hypercalcaemia
- New diagnosis of renal stones in the woman (confirmed on imaging, after starting of IMP)
- Milk alkali syndrome (hypercalcaemia, alkalosis, and biochemical evidence of renal impairment)
- Neonatal hypocalcaemia requiring treatment
- Symptomatic neonatal hypocalcaemia (usually presenting as neonatal seizures)

Reportable adverse events will be recorded in the study database, and the assessment of severity, seriousness, and relatedness to the IMP (causality) will be documented.

An AE that meets the definition of serious (i.e. results in death, is life-threatening, requires hospitalisation or prolongation of existing hospitalisation, results in persistent or significant disability or incapacity, is a congenital anomaly/birth defect, is otherwise considered medically significant by the investigator) will be reported as an SAE. SAEs will be recorded on the SAE CRF and must be signed off by the site PI and CI. The PI will also report the SAE to their own trust in accordance with local practice and to the trial office. All SAEs are reviewed by the DMC during the DMC meetings throughout the trial.

However, there are SAEs that are considered expected for the trial population. These are recorded in the case note review but are not considered to be critical to evaluations of the safety of the trial and do not need to be reported. These include the following:

### Expected maternal serious adverse events

- Pre-planned hospitalisation
- Hospitalisation for pregnancy bleeding
- Hospitalisation for the management of pregnancy loss at gestations prior to 24 weeks
- Hospitalisation for rest in pregnancy
- Hospitalisation for observation or monitoring of pregnancy.
- Hospitalisation for maternal discomfort in pregnancy
- Hospitalisation for complications of pregnancy, e.g. urinary tract infection and pyelonephritis
- A diagnosis of pre-eclampsia (this information is already being collected on the outcome CRF)
- Hospitalisation for birth (including caesarean section)
- Prolonged hospitalisation for postnatal care

### Expected neonatal serious adverse events

- Neonatal hospitalisation for sepsis
- Neonatal hospitalisation for prematurity

### Frequency and plans for auditing trial conduct {23}

The monitoring requirements for CaPE have been developed following a trial-specific risk assessment by BCTU and are documented in the trial-specific monitoring plan.

BCTU trial team staff will be in regular contact with the site research teams to check on progress and address any queries that they may have. Trial staff will check incoming consent forms and CRFs for compliance with the protocol, data consistency, missing data, and timing. Sites will be sent data clarification forms requesting missing data or clarification of inconsistencies or discrepancies.

Sites will be requested to send in copies of signed consent forms and other documentation for in-house review for all participants providing explicit consent. This is detailed in the monitoring plan.

Additional onsite monitoring may be triggered by poor CRF return, poor data quality, low SAE reporting rates, excessive number of participant withdrawals, or deviations (also defined in the monitoring plan). The site PI must permit trial-related monitoring, audits, ethical review, and regulatory inspection(s) at their site, providing direct access to source data/documents.

For this trial, we will monitor sites in accordance with the trial risk assessment and monitoring plan.

### Plans for communicating important protocol amendments to relevant parties (e.g. trial participants and ethical committees) {25}

Any substantial protocol amendments will be submitted to Medicines & Healthcare products Regulatory Agency (MHRA), Research Ethics Committee (REC), and HRA for approval as appropriate. Research teams at each site will be notified of the amendment, and, if they have no objection to the amendment, it will be implemented at the site. Online support sessions and newsletters will allow the trial team to relay information and share challenges and good practices.

### Dissemination plans {31a}

The results of this trial will be submitted for publication in a peer-reviewed journal to be communicated to healthcare professionals. We will also disseminate the information to PPI through relevant meetings and social media platforms.

## Discussion

The grant was activated in November 2020 during the Covid pandemic, which impacted many studies, including the CaPE trial. Sites were asked to prioritise COVID research initially, followed by studies that were already open, and this delayed the start of the CaPE trial. Covid also led to a reduction in the NHS workforce and Research and Development Department capacity as a result of the reallocation of research staff to cover sickness, fatigue, and staff leaving posts. This has impacted recruitment to the CaPE trial.

The calcium supplements given as IMP are an Excess Treatment Cost, which the sites can find difficult to recoup from the Clinical Research Network (CRN), as many sites may not reach the financial threshold to recoup them. This resulted in some sites declining to join the trial and others setting lower recruitment targets. However, we have overcome this barrier with the sponsor now being allowed to recoup the costs directly from the CRN.

## Trial status

The protocol is currently v2.0, 8th August 2024. The trial began recruiting on 4th August 2022, with planned recruitment until June 2025.

## Data Availability

The final dataset will be available to members of the TMG who need access to the data to undertake the final analyses. Following publication of the trial, an anonymised final trial dataset will be made available to external researchers upon request and with approval from the TMG and the BCTU data sharing committee in line with standard data-sharing practices for clinical trial data sets.

## Abbreviations

AE: Adverse event
AKI: Acute kidney injury
ALT: Alanine aminotransferase
AST: Aspartate aminotransferase
BCTU: Birmingham Clinical Trials Unit
BMI: Body mass index
CACE: Complier average causal effect
CEAC: Cost-effectiveness acceptability curve
CI: Chief investigator
CI: Confidence interval
CLD: Chronic lung disease
COS: Core outcome set
CRF: Case report form
CRN: Clinical Research Network
DIC: Disseminated intravascular coagulation
DMC: Data Monitoring Committee
FMF: Fetal Medicine Foundation
EDD: Estimated delivery date
GCP: Good Clinical Practice
HELLP: Haemolysis elevated liver enzymes low platelets
HRA: Health Research Authority
IMP: Investigational medicinal product
IPD: Individual participant data
ISSHP: International Society for the Study of Hypertension in Pregnancy
ITU: Intensive therapy unit
IVH: Intraventricular haemorrhage
MHRA: The Medicines & Healthcare products Regulatory Agency
NEC: Necrotising enterocolitis
NHS: National Health Service
NICE: National Institute for Health and Care Excellence
NNU: Neonatal unit
PCR: Protein-creatinine ratio
PI: Principal investigator
PIS: Participant information sheet
PPI: Patient and public involvement
REC: Research Ethics Committee
ROP: Retinopathy of prematurity
SAE: Serious adverse event
SAP: Statistical analysis plan
SLE: Systemic lupus erythematosus
RR: Relative risk
TMG: Trial Management Group
TSC: Trial Steering Committee
UK: United Kingdom

## References

[1] Tan MY, Wright D, Syngelaki A, et al. Comparison of diagnostic accuracy of early screening for pre-eclampsia by NICE guidelines and a method combining maternal factors and biomarkers: results of SPREE. Ultrasound Obstet Gynecol. 2018;51(6):743–50. 29536574 10.1002/uog.19039

[2] Brown MA, Magee LA, Kenny LC, et al. Hypertensive disorders of pregnancy: ISSHP classification, diagnosis, and management Recommendations for International Practice. Hypertension. 2018;72:24–43. 29899139 10.1161/HYPERTENSIONAHA.117.10803

[3] DH, editor. Confidential Enquiry into Maternal and Child Health (CEMACH). Perinatal Mortality 2006: England, Wales, and Northern Ireland. London: CEMACH; 2008.

[4] Ananth CV, Vintzileos AM. Maternal-fetal conditions necessitating a medical intervention resulting in preterm birth. Am J Obstet Gynecol. 2006;195(6):1557–63. 17014813 10.1016/j.ajog.2006.05.021

[5] Meads CA, Cnossen JS, Meher S, et al. Methods of prediction and prevention of pre-eclampsia: systematic reviews of accuracy and effectiveness literature with economic modelling. Health Technol Assess. 2008;12(6):iii–iv, 1-270. 18331705 10.3310/hta12060

[6] Redman CW, Sargent IL. Latest advances in understanding preeclampsia. Science. 2005;308(5728):1592–4. 15947178 10.1126/science.1111726

[7] Duley L, Meher S, Hunter KE, Seidler AL, Askie LM. Antiplatelet agents for preventing pre‐eclampsia and its complications. Cochrane Database Syst Rev. 2019;(10):CD004659. 10.1002/14651858.CD004659.pub3. 31684684 10.1002/14651858.CD004659.pub3 PMC6820858

[8] Rolnik DL, Wright D, Poon LC, O’Gorman N, Syngelaki A, de Paco MC, et al. Aspirin versus placebo in pregnancies at high risk for preterm preeclampsia. N Engl J Med. 2017;377(7):613–22. 10.1056/NEJMoa1704559. Epub 2017 Jun 28 PMID: 28657417. 28657417 10.1056/NEJMoa1704559

[9] Hastie R, Tong S, Wikström AK, Sandström A, Hesselman S, Bergman L. Aspirin use during pregnancy and the risk of bleeding complications: a Swedish population-based cohort study. Am J Obstet Gynecol. 2021;224(1):95.e1-95.e12. 32687818 10.1016/j.ajog.2020.07.023

[10] Hypertension in pregnancy: diagnosis and management. NICE Guideline. 2019. Available from www.nice.org.uk/guidance/ng133. Accessed 08 Dec 2020.

[11] Belizan JM, Villar J. The relationship between calcium intake and edema-, proteinuria-, and hypertension-getosis: an hypothesis. Am J Clin Nutr. 1980;33(10):2202–10. 6999886 10.1093/ajcn/33.10.2202

[12] Villar J, Belizan JM, Fischer PJ. Epidemiologic observations on the relationship between calcium intake and eclampsia. Int J Gynaecol Obstet. 1983;21(4):271–8. 6141080 10.1016/0020-7292(83)90016-4

[13] Rhj H. Prevention of pre-eclampsia. Lancet. 1962;1:864–5.

[14] Weigel MM, Narvaez WM, Lopez A, et al. Prenatal diet, nutrient intake and pregnancy outcome in urban Ecuadorian primiparas. Arch Latinoam Nutr. 1991;41(1):21–37. 1822067

[15] Belizan JM, Villar J, Repke J. The relationship between calcium intake and pregnancy-induced hypertension: up-to-date evidence. Am J Obstet Gynecol. 1988;158(4):898–902. 3284363 10.1016/0002-9378(88)90091-9

[16] Carroli G, Merialdi M, Wojdyla D, et al. Effects of calcium supplementation on uteroplacental and fetoplacental blood flow in low-calcium-intake mothers: a randomized controlled trial. Am J Obstet Gynecol. 2010;202(1):45 e1-9. 19716540 10.1016/j.ajog.2009.07.037

[17] Hofmeyr GJ, Lawrie TA, Atallah ÁN, Torloni MR. Calcium supplementation during pregnancy for preventing hypertensive disorders and related problems. Cochrane Database Syst Rev. 2018;(10):CD001059. 10.1002/14651858.CD001059.pub5. 10.1002/14651858.CD001059.pub5 PMC6517256 30277579

[18] Cormick G, Betrán AP, Romero IB, Lombardo CF, Gülmezoglu AM, Ciapponi A, et al. Global inequities in dietary calcium intake during pregnancy: a systematic review and meta-analysis. BJOG. 2019;126(4):444–56. 10.1111/1471-0528.15512. 30347499 10.1111/1471-0528.15512 PMC6518872

[19] De Souza E, Sass N. Aspirin and calcium to prevent preeclampsia in chronic hypertensive women with abnormal uterine artery Doppler ultrasound. Rev Bras Ginecol Obstet. 2006;28(2):136.

[20] Snell KIE, Allotey J, Smuk M, et al. External validation of prognostic models predicting pre-eclampsia: individual participant data meta-analysis. BMC Med. 2020;18(1):302. 33131506 10.1186/s12916-020-01766-9 PMC7604970

[21] Fetal Medicine Foundation Algorithm: Risk Assessment for Pre-eclampsia, available from https://fetalmedicine.org/research/assess/preeclampsia/first-trimester. Accessed 10 May 2021.

[22] Poon LC, Wright D, Thornton S, et al. Mini-combined test compared with NICE guidelines for early risk-assessment for pre-eclampsia: the SPREE diagnostic accuracy study. Southampton (UK): NIHR Journals Library; 2020. PMID: 33226739. 33226739

[23] BNF: National Institute of Clinical Excellence. Available from: https://bnf.nice.org.uk/interaction/calcium-carbonate-2.html . Accessed 15 May 2018.

[24] Villar J, Repke J, Belizan JM, et al. Calcium supplementation reduces blood pressure during pregnancy: results of a randomized controlled clinical trial. Obstet Gynecol. 1987;70(3 Pt 1):317–22. 3306493

[25] Dwarkanath P, Muhihi A, Sudfeld CR, Wylie BJ, Wang M, Perumal N, et al. Two randomized trials of low-dose calcium supplementation in pregnancy. N Engl J Med. 2024;390(2):143–53. 10.1056/NEJMoa2307212. 38197817 10.1056/NEJMoa2307212 PMC10921922

[26] US Department of Health and Human Services. National Institutes of Health, Office of Dietary Supplements. Calcium Fact Sheet for Professionals. Available from: https://ods.od.nih.gov/factsheets/Calcium-HealthProfessional/. Accessed 3 Jul 2018.

[27] Levine RJ, Hauth JC, Curet LB, Sibai BM, Catalano PM, Morris CD, et al. Trial of calcium to prevent preeclampsia. N Engl J Med. 1997;337(2):69–76. 9211675 10.1056/NEJM199707103370201

[28] World Health Organization Recommendations for Prevention and Treatment of Pre-eclampsia and Eclampsia. Available from: http://apps.who.int/iris/bitstream/handle/10665/44703/9789241548335_eng.pdf;jsessionid=F334CA. Accessed 1 Aug 2018. 23741776

[29] Askie LM, Duley L, Henderson-Smart DJ, et al. Antiplatelet agents for prevention of pre-eclampsia: a meta-analysis of individual patient data. Lancet. 2007;369(9575):1791–8. 17512048 10.1016/S0140-6736(07)60712-0

[30] Meher S, Walkinshaw S, Neilson J. Risk of pre-eclampsia in women with two moderate risk factors. Archives of Disease in Childhood-Fetal and Neonatal Edition 2011; 96 (Supp 1): Fa102-Fa103.

[31] Tucker KL, Mort S, Yu LM, et al. Effect of self-monitoring of blood pressure on diagnosis of hypertension during higher-risk pregnancy. JAMA. 2022;327(17):1656. 35503346 10.1001/jama.2022.4712 PMC9066279

[32] Macdonald HM, Garland A, Burr J, Strachan A, Wood AD, Jamil NA, et al. Validation of a short questionnaire for estimating dietary calcium intakes. Osteoporos Int. 2014;25(6):1765–73. 10.1007/s00198-014-2694-5. 24668005 10.1007/s00198-014-2694-5

[33] CGEM Calcium Calculator. Available from: https://webapps.igc.ed.ac.uk/world/research/rheumatological/calcium-calculator/.

[34] NICE. NICE health technology evaluations: the manual—economic evaluation. NICE; 2022 [updated 2025 Jul 14; cited 2025 Sep 5]. Available from: https://www.nice.org.uk/process/pmg36/chapter/economic-evaluation-2

[35] Ramsey SD, Willke RJ, Glick H, Reed SD, Augustovski F, Jonsson B, et al. Cost-effectiveness analysis alongside clinical trials II-an ISPOR good research practices task force report. Value Health. 2015;18(2):161–72. 10.1016/j.jval.2015.02.001. 25773551 10.1016/j.jval.2015.02.001

[36] Husereau D, Drummond M, Augustovski F, de Bekker-Grob E, Briggs AH, Carswell C, et al. Consolidated health economic evaluation reporting standards (CHEERS) 2022 explanation and elaboration: a report of the ISPOR CHEERS II good practices task force. Value Health. 2022;25(1):10–31. 10.1016/j.jval.2021.10.008. 35031088 10.1016/j.jval.2021.10.008

